# A Sensitive and Rapid UPLC-MS/MS Method for Determination of Monosaccharides and Anti-Allergic Effect of the Polysaccharides Extracted from *Saposhnikoviae Radix*

**DOI:** 10.3390/molecules23081924

**Published:** 2018-08-01

**Authors:** Yan-Yan Gao, Yue Jiang, Guo-Chao Chen, Shuang-Shuang Li, Fei Yang, Qun Ma

**Affiliations:** School of Chinese Materia Medica, Beijing University of Chinese Medicine, Beijing 102488, China; lhshjyfj@163.com (Y.-Y.G.); hydrationjy@163.com (Y.J.); chenguochaocgc@163.com (G.-C.C.); 18801378710@163.com (S.-S.L.); yf18910268413@163.com (F.Y.)

**Keywords:** anti-allergic effect, composition, polysaccharides, *Saposhnikoviae Radix*, UPLC-MS/MS

## Abstract

**Background**: Allergic disease is a common clinical disease. Natural products provide an important source for a wide range of potential anti-allergic agents. This study was designed to evaluate the anti-allergic activities of the water-soluble polysaccharides extracted and purified from *Saposhnikoviae Radix* (SRPS). The composition and content of monosaccharides were determined to provide a material basis. **Methods**: An ultra-high-performance liquid chromatography-tandem mass spectrometry (UPLC-MS/MS) method was established to determine the composition and content of SRPS. 2,4-dinitrofluorobenzene (DNFB) induced a delayed-type hypersensitivity (DTH) mouse model orally administrated SRPS for seven consecutive days. Ear swelling, organ index, and serum IgE levels were observed to evaluate the anti-allergic activities. **Results**: The UPLC-MS/MS analysis showed that SRPS was consisted of eight monosaccharides including galacturonic acid, mannose, glucose, galactose, rhamnose, fucose, ribose, and arabinose with a relative molar ratio of 4.42%, 7.86%, 23.69%, 12.06%, 3.10%, 0.45%, 0.71%, and 47.70%, respectively. SRPS could effectively reduce ear swelling, a thymus index, and a serum IgE levels. **Conclusions**: The method was simple, rapid, sensitive, and reproducible, which could be used to analyze and determine the monosaccharide composition of SRPS. The vivo experiments demonstrated that SRPS may effectively inhibit development of DNFB-induced DTH. SRPS is a novel potential resource for natural anti-allergic drugs.

## 1. Introduction

Allergic diseases may occur from newborns to the elders at all ages, which are a common clinical disease [1,2,3]. Most patients cannot find the exact cause due to their complex causes of disease, which lacks specific therapeutic drugs. At present, the main treatment for allergic diseases is to avoid allergens and to use a variety of anti-inflammatory substances [4]. However, most of them (e.g., hormones) have a considerable side effect such as dizziness, drowsiness, nausea, and more even though they have a higher level of therapeutic effect [5]. In the face of the high incidence of allergic diseases, the existing drugs are not enough to meet treatment needs. It is urgent to develop new highly effective drugs with little side effect. Natural products have the characteristics of mild efficacy, low toxicity, side effects, and multiple targets and provide an important source for a wide range of potential new agents [6,7,8,9].

*Saposhnikoviae Radix* is the dried root of the umbrella plant *Saposhnikovia divaricata* (Turcz.) Schischk. It has the functions of expelling wind, relieving pain, and relieving spasms, which is used for cold headache, rheumatism, and rubella itch [10]. *Saposhnikoviae Radix* is the preferred drug to treat pruritus caused by various reasons in the clinic. Pharmacological studies have shown that *Saposhnikoviae Radix* has antipyretic, analgesic, anti-inflammatory, antibacterial, anti-tumor, and other biological activities [11]. Wu et al. reported that the *Saposhnikoviae Radix* extract could inhibit mast cell degranulation by reducing the expression of PAR-2, which eventually resulted in an anti-allergic effect [12]. Zhao et al. reported that chromone from *Saposhnikoviae Radix* contributed to an amelioration of arthritis severity in collagen II-induced arthritis. The mechanism might be related to the inhibited activities of the pro-inflammatory cytokines [13].

The above studies indicate that the *Saposhnikoviae Radix* is a safe and effective anti-allergic agent. However, at present, the research on the anti-allergic activity of *Saposhnikoviae Radix* only use the crude alcohol extract, the volatile oil, or the decoction for the experimental study of drug efficacy. Most notably, the existing quality control indicators cannot objectively reflect a material basis. An anti-allergic effect and mechanism of the polysaccharides extracted from *Saposhnikoviae Radix* (SRPS) are not clear. Moreover, different biological activities are closely related to the composition of polysaccharides. It is very necessary for establishing a sensitive and rapid method for the determination of monosaccharides. In view of this, SRPS was extracted and purified and its monosaccharide composition and content was analyzed using UPLC-MS/MS for the first time. Then effects of SRPS on allergy were evaluated with 2,4-dinitrofluorobenzene (DNFB) induced a delayed-type hypersensitivity (DTH) mouse model. This study aims to reveal the corresponding relationship between the anti-allergy pharmacological effects of SRPS and the material basis to provide a reference for clinical medication.

## 2. Results 

### 2.1. Determination of Monosaccharide Composition and Content in SRPS

#### 2.1.1. UPLC-MS/MS Conditions

**Figure 1:**
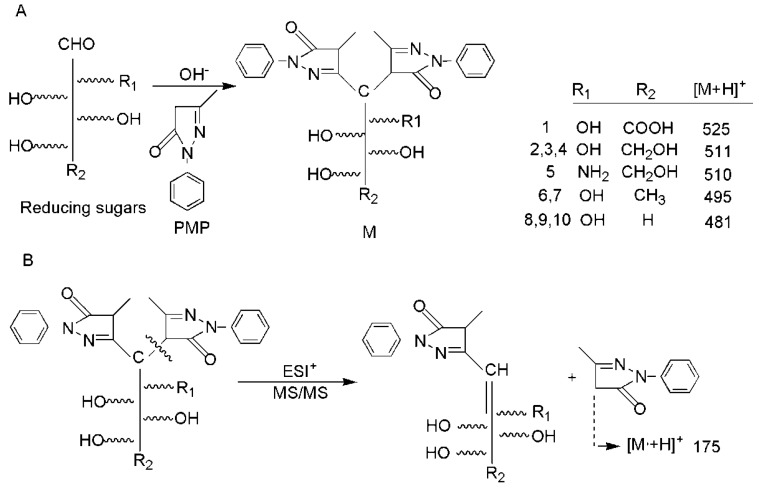
(**A**) Reaction of PMP reagents with reducing monosaccharides: 1, galacturonic acid (Gal-A), 2, mannose (Man), 3, glucose (Glu), 4, galactose (Gal), 5, IS, Glucosamine (D-Glu), 6, rhamnose (Rha), 7, fucose (Fuc), 8, Ribose (Rib), 9, arabinose (Ara), and 10, xylose (Xyl); (**B**) The typical ESI^+^ fragmentation pathway.

**Figure 2:**
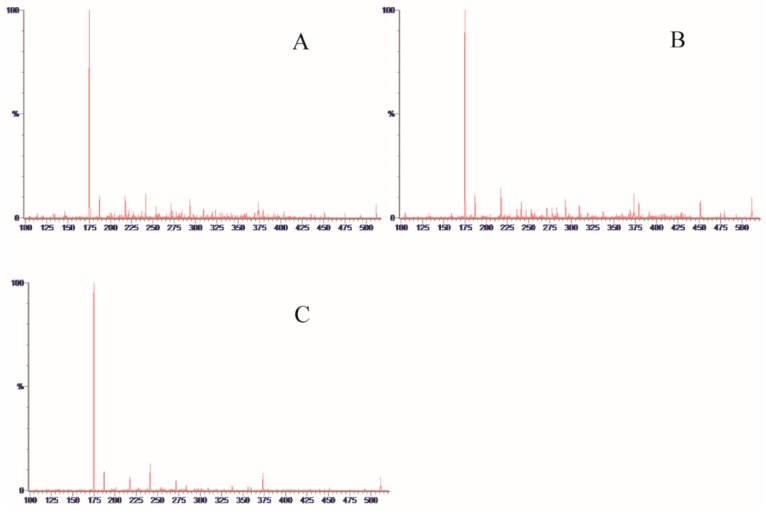
MS^2^ spectra of PMP-labeled: (**A**), Mannose; (**B**) Glucose; and (**C**) Galactose.

The determination of the monosaccharide composition of polysaccharides is the most important part to control polysaccharide quality, which also provides basic information in polysaccharides. The derivatization method is commonly used in chromatography detection due to monosaccharides that have special structures and lack optical activity. 1-Pheny-3-Methyl-5-Pyrazolone (PMP) derivatization reaction conditions are mild and can react quantitatively with reducing sugars, which was first applied to a polysaccharide analysis in 1989 [14]. In this study, an improved pre-column derivatization method for PMP was used, which eliminated the desalination process prior to mass spectrometry [15]. Most notably, the improved method was applied to analyze SRPS for the first time by UPLC-MS/MS. The PMP reaction mechanism was illustrated in Figure 1A. The typical ESI^+^ fragmentation pathway was illustrated in Figure 1B. When taking the channel of 511.259/175.123 as an example, the *m*/*z* 175 has a strong correspondence obtained in MS^2^, which could be used to confirm a monosaccharide with a positive ion mode (Figure 2).

**Figure 3:**
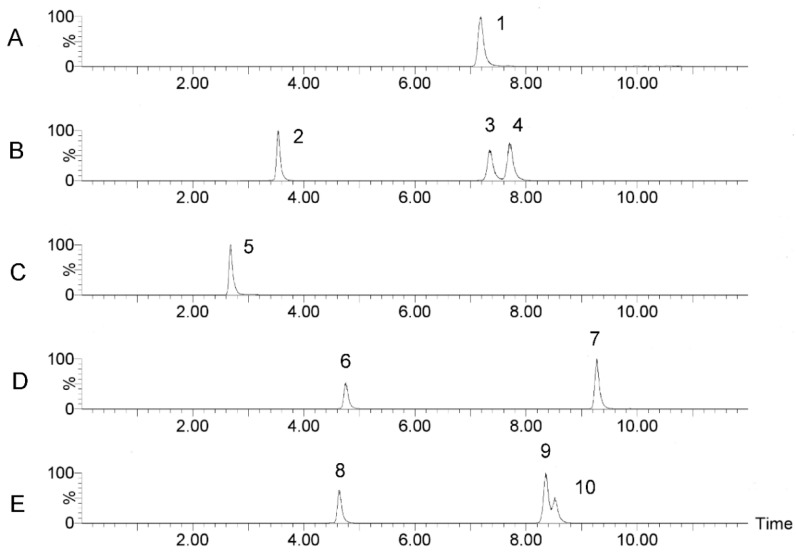
MRM chromatograms of standards: (**A**) 1, Gal-A; (**B**) 2, Man, 3, Glu, 4, Gal; (**C**) 5, Glu-N; (**D**) 6, Rha, 7, Fuc; (**E**) 8, Rib, 9, Ara, and 10, Xyl.

**Figure 4:**
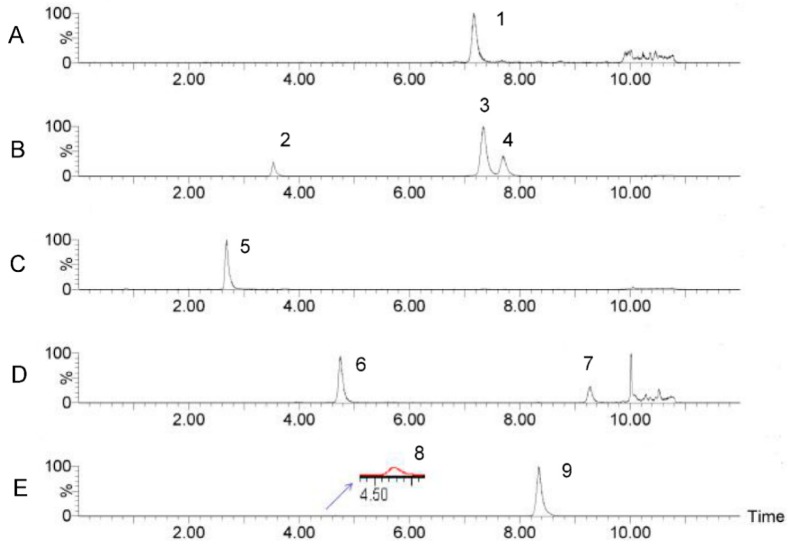
MRM chromatograms of SRPS: (**A**) 1, Gal-A; (**B**) 2, Man, 3, Glu, 4, Gal; (**C**) 5, Glu-N; (**D**) 6, Rha, 7, Fuc; (**E**) 8, Rib, 9, Ara.

Lastly, optimized mass spectrometry analysis parameters of the 10 monosaccharides were shown in Table 1. The advantage of the current method in comparison with traditional HPLC-UV methods is that it could effectively eliminate the interference of the PMP ion peak using UPLC-MS/MS with MRM detection. A representative multiple reaction monitoring (MRM) chromatogram of a mixed standard solution of nine monosaccharides and IS (glucosamine) separated under the optimized chromatography and detection conditions was shown in Figure 3. A representative MRM chromatogram of SRPS was shown in Figure 4.

#### 2.1.2. Quantification and Validation

The calibration curve for each compound was obtained with at least six appropriate concentrations. The regression equations for the nine monosaccharides were calculated in the form of y = ax + b where y and x were the peak area and the corresponding sample content of monosaccharides was injected, respectively. As shown in Table 2, the established method showed good linearity and the correlation coefficients of all target components exceeded 0.9970. The limits of detection (LODs) and lower limits of quantitation (LLOQs) under the present chromatographic conditions were determined at a signal-to-noise ratio (S/N) of 3 and 10 by series dilution from stock solution with LODs ranged from 1.50 ng/mL to 2.49 ng/mL and LLOQs ranged from 4.94 ng/mL to 8.21 ng/mL, respectively.

For the precision test, the mixed standard solutions were analyzed for six replicates within a day. The measured peak area was brought into line with the standard curve of the day to calculate the concentration of the mixed reference solution. The coefficient of variation (RSD) was used to investigate the closeness of the assay (Precision). The RSD of content for each compound was not more than 2.66%. The 40, 80, 120 μM mixed reference solution was prepared and analyzed according to the optimal conditions. Three parallel samples were prepared at each concentration and examined in three batches. For each concentration level, recovery was calculated by comparing the average of nine measurements and the theoretical added value, which indicates the accuracy of the method. The recoveries of analytes varied from 94.65% to 115.80%. Six samples of SRPS were prepared. The measured peak area was taken into the standard curve of the day to calculate the concentration of each monosaccharide in the sample solution. The repeatability of the determination results was analyzed by the coefficient of variation (RSD). The results were found to be within the acceptance criteria. The results were shown in Table 3.

#### 2.1.3. Determination of Monosaccharide Composition and Content in SRPS

The established UPLC-MS/MS method was used to analyze SRPS samples under optimized conditions. The analysis results of SRPS were listed in Table 4. The samples were measured three times in parallel and the results were expressed as mean values. UPLC-MS/MS analysis showed that the polysaccharides consisted of eight monosaccharides including galacturonic acid, mannose, glucose, galactose, rhamnose, fucose, ribose, and arabinose with a relative molar ratio of 4.42%, 7.86%, 23.69%, 12.06%, 3.10%, 0.45%, 0.71%, and 47.70%, respectively. The content of arabinose in the polysaccharides was the highest and accounts for more than 40% of the total sugar content, which is followed by glucose. No xylose was detected in SRPS.

### 2.2. The Effect of SRPS on Anti-Allergic Effect

#### 2.2.1. The Effect of SRPS on Ear Swelling

Contact dermatitis is a form of delayed-type hypersensitivity characterized by localized thickening, papules, redness, and vesicles of the skin. A model of contact dermatitis involving a repeated challenge with a half antigen is adapted to assess dermatitis as characterized by skin thickening. Ear swelling rate is one of the important indicators [16,17,18]. As shown in Table 5, there was no significant change in the weight of the left and right ears in the normal group while the weight in the right ears of the model group was significantly increased when compared with the normal group (*p* < 0.01), which indicates that the DNFB-induced DTH mouse model was established successfully. When administered orally for seven days immediately before the first DNFB challenge in the ear, SRPS significantly inhibited ear swelling by 16.90%, 36.78%, and 49.01% at the dose of 80 mg/kg BW, 120 mg/kg BW, and 160 mg/kg BW, respectively (*p* < 0.05).

#### 2.2.2. The Effect of SRPS on the Body Weight and the Relative Weight of the Organ

**Figure 5:**
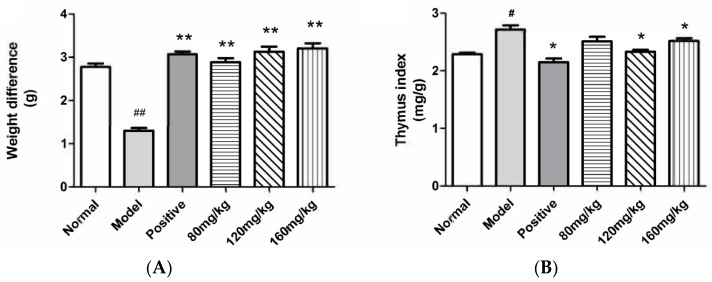
(**A**) Effect of SRPS on body weight; (**B**) Effect of SRPS on thymus. (Values are means ± SE. (n = 6). ^#^ *p* ≤ 0.05, ^##^ *p* ≤ 0.01 vs. normal control. * *p* ≤ 0.05, ** *p* ≤ 0.01 vs. model control).

The body weights of the treated animals were recorded in Figure 5A. Compared with the normal group, the body weight of the model group was significantly reduced. SRPS could effectively reduce the weight loss of mice caused by DTH (*p* < 0.05). As shown in Table 6, there was no significant difference between the spleen index, the kidney index, and the adrenal index in the treatment group and the control group. Thymus is an important central immune organ participated in cellular immunity where T cells differentiate and develop [19]. Compared with a DTH model group, the high and middle dose group could effectively reduce the thymus index of DTH mice. The difference was statistically significant (*p* < 0.05). The date was shown in Figure 5B. The results showed that SRPS could inhibit cellular immunity.

#### 2.2.3. Determination of Serum Total IgE

**Figure 6:**
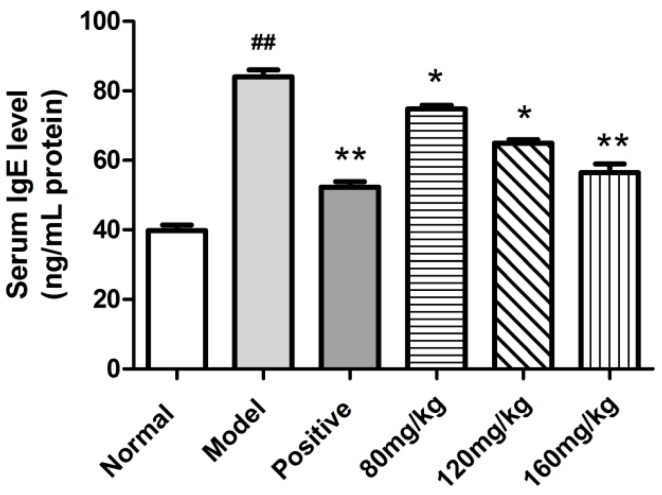
Serum IgE concentrations in the indicated groups measured 24 h after the last DNFB challenge. (Values are means ± SE. (n = 6). ^#^ *p* ≤ 0.05, ^##^ *p* ≤ 0.01 vs. normal control. * *p* ≤ 0.05, ** *p* ≤ 0.01 vs. model control).

The serum IgE levels in DNFB-treated mice were measured. As shown in Figure 6, serum IgE levels in the SRPS treated mice (75.79 ng/mL at the dose of 80 mg/kg) decreased significantly compared to the mice in the model group (84.01 ng/mL) (*p* < 0.05). Particularly at the dose of 160 mg/kg, SRPS displayed a more potent reduction in the level of serum IgE. 

## 3. Discussion

In traditional Chinese medicine, *Saposhnikoviae Radix*, which is a famous herb, has been widely and successfully used for many years. In a previous study, the aqueous extract of *Saposhnikoviae Radix* has been shown to have immune activity [20]. However, the material basis is not clear. Polysaccharides have many different kinds of biological activities. The immune activity is recognized as the remarkable feature and it can modulate the body’s immune functions by regulating immune organs, immune cells, and immune molecules [21,22,23]. Therefore, in this study, we attempted to clarify the effective component of the *Saposhnikoviae Radix* aqueous extract. The water-soluble polysaccharides were extracted and purified from the *Saposhnikoviae Radix* to evaluate the anti-allergic activities. The change of the immune organ index is one of the typical symptoms in mice with allergy. The improvement of the immune organ index indicated that the allergy of mice has been mitigated. The thymus is an important organ of the immune system in the body [24,25]. Compared to the model group, the thymus index of SRPS-treated groups has been improved (Figure 5). Erythema, eczema, and pruritus with atopic dermatitis are closely linked to elevated serum lgE levels. It has been known that mast cell activation and their histamine release were tightly modulated by IgE from B cells [26,27,28]. Therefore, we measured the serum IgE levels in DNFB-treated mice. As shown in Figure 6, SRPS displayed a potent reduction in the level of serum IgE. Th1/Th2 maintains normal homeostasis in a normal body. When Th2 is excessive, its secreted IF-4 can promote the proliferation of B cells and induces the production of antibodies especially IgE, which causes an allergic reaction [29]. SRPS could significantly reduce serum IgE levels, which may be related to the control of various Th1 and Th2 related factors. However, its relevant mechanism needs further study. Furthermore, no toxicity was observed in thymus, kidney, spleen, and the adrenal gland after SRPS treatment. SRPS could reduce the weight loss of mice induced by DTH.

In addition, biological activity is closely related to the composition and content of polysaccharides. Therefore, in this study, we successfully established a pre-column derivatization with a UPLC-MS/MS method for simultaneous determination of eight monosaccharides in SRPS. Compared with the traditional monosaccharide composition analysis methods, the method was simple, rapid, sensitive, and reproducible. UPLC-MS/MS analysis showed that the content of arabinose in the polysaccharides was the highest, which accounted for more than 40% of the total sugar content followed by glucose and galactose (Table 4). Georgiev et al. reported that the chemical features of the three *purslane* polysaccharide complexes such as the highest galactose, arabinose, and glucose contents were important in immunomodulating activity [30,31,32]. Akhtar et al. reported that arabinose contained in wheat bran could enhance humoral immune function and significantly increase serum antibody levels in animal bodies [33]. The anti-allergic activity of SRPS might be closely related to its high content of arabinose. However, in addition to the monosaccharide composition, the immunoregulatory activity of polysaccharides is also related to its structure and its molecular weight [34]. Further research is needed in this regard.

In summary, the above results indicated that SRPS has an anti-allergic activity and could be used as a potential drug to treat allergic diseases. Our results laid the foundation for the quality control and activity study of the *Saposhnikoviae Radix* and provided a scientific basis for the discovery of new potential active drugs against allergic diseases.

## 4. Materials and Methods

### 4.1. Chemicals and Reagents

*Saposhnikoviae Radix* was purchased in Beijing Sanhe Pharmaceutical Co. Ltd. (Beijing, China). D-Glucose, D-arabinose, and other standards were purchased from Shanghai Bio-Technology Co. Ltd. (Shanghai, China) (purity > 98.0%). DNFB was purchased from Macklin Biochemical Technology Co. Ltd. (Shanghai, China). Mouse IgE Elisa Kit was produced by Bio-Swamp Life Science Lab (Wuhan Myhalic Biotechnology Co. Ltd., Wuhan, China). In addition, acetonitrile, methanol, and ammonium acetate were chromatographically pure and all other chemicals and reagents were of analytical grade.

### 4.2. Chemistry

#### 4.2.1. Preparation of Calibration Standards

The stock solutions of galacturonic acid, mannose, glucose, galactose, rhamnose, fucose, ribose, arabinose, xylose, and the IS (glucosamine) were individually prepared in ammonia. The stock solutions of the standards were further diluted in ammonia to produce combined standard working solutions at a series of concentrations. The IS solution (107.8 ng/mL) was obtained by diluting the stock solution in ammonia. 

#### 4.2.2. Extraction and Purification of SRPS

A total of 6 L of water was added to 600 g *Saposhnikoviae Radix* slices extracted three times for 1.5 h. The extract was concentrated to a crude drug content of 1 g/mL, which added ethanol to precipitate that the final ethanol concentration was 80%. The supernatant was removed by standing filtration and the precipitate was dried to obtain a crude extract of polysaccharides. The crude extract was dissolved in 200 mL of distilled water. Protein was dislodged by the Sevag method [35]. The protein-removing polysaccharides were dissolved in 30 mL distilled water and then the solution applied to a D101 macro-porous resin-column (40 cm × 2.6 cm). The column was first eluted with distilled water of 1 bed volume to remove small molecular impurities. The fractions obtained were combined according to the 1-naphthol reaction with the formation of a purple ring. The combined eluate was precipitated with ethanol, filtered, and the precipitate was dried in a vacuum oven to obtain the purified polysaccharides. The total sugar content of polysaccharides was 58.93% with a method of Phenol-sulfuric acid. 

#### 4.2.3. Hydrolysis and Derivatization of Polysaccharides

A total of 10 mg of polysaccharides were added to 2 mL 2 M trifluoroacetic acid in a boiling water bath for 8 h and cooled to room temperature. The reaction mixture was centrifuged 8000 r/min for 15 min. The supernatant was evaporated to dryness. After 50 μL of IS work solution (107.8 ng/mL) was added to the mixed standard solution (50 μL), 100 μL of PMP was added (0.5 M). The mixture was vortex-mixed for 2 min and placed in a 70 °C water bath for 30 min. Then the sample was added to an appropriate amount of acetic acid and extracted three times with dichloromethane with 2 mL each. The upper solution after extraction was placed in a centrifuge tube centrifuged at 13,000 r/min 10 min. The supernatant was collected. The monosaccharides of the polysaccharides hydrolyzed by the trifluoroacetic acid were dissolved in ammonia water of 1 mL. The same derivatization process was performed as described above in the monosaccharide standard derivatization step [36]. Lastly, 2 μL of the supernatant was injected for UPLC-MS/MS analysis.

#### 4.2.4. Chromatographic and Mass Spectrometry Conditions

The mobile phases were composed of 0.5 mM ammonium acetate and 0.05% acetic acid buffer solution (A) and acetonitrile (B) at a flow rate of 0.4 mL/min. Using a gradient started at 18% to 19% B at 0–1.0 min, 19% to 20% B at 1.0–4.0 min, 20% to 21% B at 4.0–7.0 min, 21% to 25% B at 7.0–9.0 min, 95% to 18% B at 10.0–10.1 min, 18% B at 10.1–12.0 min. After optimizing the chromatographic conditions, the column temperature was adjusted to 30 °C and the autosampler was conditioned at 10 °C. A total of 2 μL of the sample or standard solution was injected into the system for analysis.

The analytes were detected using MRM in a positive ionization mode. Capillary voltage was 3.0 kV, ion source temperature was 150 °C, and desolvation gas (nitrogen) temperature was 400 °C. The flow rate of the nebulizer gas (nitrogen) was 800 L/h, the flow rate of the cone gas (nitrogen) was 150 L/h, the flow rate of the collision gas was 0.13 mL/min, and dwell time was 0.025 s. 

### 4.3. Anti-Allergic Activity of SRPS

#### 4.3.1. Animals

The Institute of Cancer Research (ICR) female mice (weighing 20 ± 2 g, Grade II) were obtained from the SPF Biotechnology Co. Ltd. (Beijing, China). Animals were tested by the Chinese People’s Liberation Army Academy of Military Medical Laboratory Animal Center (Certificate No. 11401500028791). Pathogen-free feed and water was supplied to the mice and the room temperature was maintained at 22 ± 1 °C and submitted to a normal light/dark (12 h/12 h) cycle. In this study, the procedures related to animal care were performed in accordance with the internationally accepted principles as listed in the Guidelines for Keeping Experimental Animals issued by the government of China. The mice were randomly divided into six groups (n = 6 for each group) as follows: (1) normal control group; (2) model group; (3) positive control group (Prednisolone 3 mg/kg body weight (BW)) and three SRPS administration groups (80, 120, 160 mg/kg BW).

#### 4.3.2. DNFB-Induced DTH and Treatment with SRPS in Experimental Animals

The abdomen of the ICR mice was shaved and uniformly coated with 30 μL of 0.5% DNFB solution (dissolved in 4:1 acetone/olive oil) in the range of 1 × 1 cm^2^. The blank group was smeared with acetone/olive oil solvent one time a day for two days. After the second sensitization, the challenge test was carried out for five days. The back of the right ear of the mice was evenly smeared with 10 μL of 0.2% DNFB solution. To the first abdominal sensitization began continuous administration for seven days [37,38,39]. SRPS (80 mg/kg BW, 120 mg/kg BW, and 160 mg/kg BW) or prednisolone (positive control, 3 mg/kg BW) suspended in normal saline was administered orally once a day. The normal control group and the model group were treated with 0.2 mL of normal saline for the same way.

#### 4.3.3. Determination of Indicators

The weights of mice were measured after 24 h of the DNFB challenge on the right ear. After the mice were anesthetized with 0.1 mL 10% chloral hydrate solution and the eyeballs were taken for blood collection. Then both ears were cut down. A diameter of 8 mm puncher removed left and right ears and weighed. The mice were killed by cervical vertebrae dislocation and four organs (thymus, spleen, renal, adrenal gland) were excised. The weights of the organs were measured.

After blood was collected from the sacrificed mice, serum samples were obtained by clotting for 30 min before centrifugation for 15 min at approximately 1000 r/min and were stored at −80 °C until use. Mouse serum IgE concentration was determined using a Mouse IgE Elisa Kit (Bio-Swamp), according to the manufacturer’s instructions.

### 4.4. Statistical Analysis

All the data was processed by statistical software SPSS 17.0, expressing as mean ± SE. The statistical significance was determined by using the Student’s *t*-test. A *p* value less than 0.05 was considered statistically significant.

## Figures and Tables

**Table 1:** Mass spectrometry analysis parameters of monosaccharides.

No.	Compound Name	t_R_ (min)	Q_1_	Q_2_	CV	CE
**1**	Gal-A	7.75	525.223	175.125	22	22
**2**	Man	3.84	511.259	175.123	6	24
**3**	Glu	7.84	511.259	175.123	28	22
**4**	Gal	8.20	511.259	175.123	36	22
**5**	D-Glu	3.04	510.323	175.123	20	32
**6**	Rha	5.16	495.312	175.124	30	22
**7**	Fuc	9.64	495.312	175.124	26	20
**8**	Rib	5.04	481.169	175.129	26	22
**9**	Ara	8.80	481.169	175.129	26	22
**10**	Xyl	8.96	481.169	175.129	4	22

**Table 2:** Summarization of calibration results, LOD, and LOQ values.

Compound Name	Regression Equation	Linear Range (ng/mL)	Correlation Coefficient	LODs (ng/mL)	LOQs (ng/mL)
Gal-A	y = 4439.12x − 1286.93	4.85–194	0.9972	2.49	8.21
Man	y = 5646.84x + 859.335	4.50–180	0.9992	2.04	6.74
Glu	y = 1401.36x + 35412.6	4.50–540	0.9980	1.95	6.43
Gal	y = 319.006x + 48035.9	4.50–540	0.9998	2.02	6.67
Rha	y = 3945.84x + 2279.57	4.10–164	0.9988	1.85	6.12
Fuc	y = 3619.60x − 1191.29	4.10–164	0.9988	1.66	5.49
Rib	y = 5312.12x − 3594.90	3.75–150	0.9970	1.74	5.74
Ara-Xyl	y = 5840.51x + 111844	3.75–750	0.9980	1.50	4.94

Note: LOD (ng/mL) = 3 × C (ng/mL)/(S/N), LLOQ (ng/mL) = 3.3 × LOD.

**Table 3:** Summary of precision, repeatability, and recovery.

Compound Name	Precision (n = 6)	Repeatability (n = 6)	Recovery (n = 3)		
	RSD (%)	RSD (%)	40 μM (%)	80 μM (%)	120 μM (%)
Gal-A	2.37	2.85	104.07	96.52	94.65
Man	1.88	2.71	113.11	102.19	102.39
Glu	1.24	2.28	102.38	100.76	103.20
Gal	2.15	3.37	102.59	101.08	100.16
Rha	1.09	2.68	115.80	113.18	105.25
Fuc	2.08	4.44	105.12	108.64	107.50
Rib	2.66	2.99	114.43	108.80	103.50
Ara-Xyl	2.09	2.09	114.58	111.30	103.19

**Table 4:** Analysis results of SRPS.

Compound Name	Content (ng/mL)	Quality Percentage	Molar Percentage
Gal-A	85.088	5.18%	4.42%
Man	140.336	8.55%	7.86%
Glu	423.062	25.76%	23.69%
Gal	215.414	13.12%	12.06%
Rha	50.411	3.07%	3.10%
Fuc	7.396	0.45%	0.45%
Rib	10.597	0.65%	0.71%
Ara	709.718	43.22%	47.70%

**Table 5:** Effect of SRPS on the ear thickness.

Groups	Dose (mg/kg)	Administration Route	Swelling (%)	Inhibition (%)
Normal	-	Oral	2.82 ± 2.20	-
Model	-	Oral	90.32 ± 3.39 ^##^	-
Positive	3	Oral	36.56 ± 2.12	59.52
	80	Oral	75.06 ± 3.82 **	16.90
SRPS	120	Oral	57.10 ± 3.19 **	36.78
	160	Oral	46.06 ± 3.13 **	49.01

Note: Each value represents the mean ± SE (n = 6) (^#^ *p* < 0.05, ^##^ *p* ≤ 0.01, significantly different from the normal control group; * *p* < 0.05, ** *p* ≤ 0.01, significantly different from the model control group).

**Table 6:** Effect of SRPS on spleen, renal, and adrenal.

Groups	Dose (mg/kg)	Spleen Index (mg/kg)	Kidney Index (mg/g)	Adrenal Index (mg/g)
Normal	-	3.11 ± 0.17	5.72 ± 0.31	0.124 ± 0.006
Model	-	3.05 ± 0.18	5.71 ± 0.29	0.127 ± 0.010
Positive	3	3.07 ± 0.18	5.65 ± 0.34	0.123 ± 0.008
	80	3.03 ± 0.24	5.70 ± 0.24	0.129 ± 0.008
SRPS	120	3.08 ± 0.25	5.68 ± 0.16	0.121 ± 0.007
	160	3.12 ± 0.13	5.69 ± 0.29	0.128 ± 0.016

Note: Each value represents the mean ± SE (n = 6).

## Abbreviations

SRPS	Polysaccharides extracted from the *Saposhnikoviae Radix*
UPLC-MS/MS	Ultra-high-performance liquid chromatography-tandem mass spectrometry
DNFB	2,4-dinitrofluorobenzene
DTH	Delayed-type hypersensitivity
IS	Internal standard
PMP	1-Pheny-3-Methyl-5-Pyrazolone
MRM	Multiple reaction monitoring
LOD	Limit of detection
LLOQ	Lower limits of quantitation
S/N	Signal-to-noise
BW	Body weight
